# Targeting CSF-1 signaling between tumor cells and macrophages at TMEM doorways inhibits breast cancer dissemination

**DOI:** 10.1038/s41388-025-03485-y

**Published:** 2025-07-11

**Authors:** Camille L. Duran, Chinmay R. Surve, Xianjun Ye, Xiaoming Chen, Yu Lin, Allison S. Harney, Yarong Wang, Ved P. Sharma, E. Richard Stanley, Dianne Cox, John C. McAuliffe, David Entenberg, Maja H. Oktay, John S. Condeelis

**Affiliations:** 1 Integrated Imaging Program for Cancer Research, Albert Einstein College of Medicine, Bronx, NY USA; 2 Department of Pathology, Albert Einstein College of Medicine, Bronx, NY USA; 3 Montefiore Einstein Comprehensive Cancer Center, Albert Einstein College of Medicine, Bronx, NY USA; 4 Cancer Dormancy and Tumor Microenvironment Institute, Albert Einstein College of Medicine, Bronx, NY USA; 5 Gruss-Lipper Biophotonics Center, Albert Einstein College of Medicine, Bronx, NY USA; 6 Bio-Imaging Resource Center, The Rockefeller University, New York, NY USA; 7 Department of Developmental and Molecular Biology, Albert Einstein College of Medicine, Bronx, NY USA; 8 Department of Surgery, Albert Einstein College of Medicine, Bronx, NY USA; 9 Department of Cell Biology, Albert Einstein College of Medicine, Bronx, NY USA; 10 Present Address: Cogent Biosciences, Waltham, MA USA

**Keywords:** Breast cancer, Cancer microenvironment, Metastasis

## Abstract

Tumor cell intravasation is essential for metastatic dissemination, but its exact mechanism is incompletely understood. We have previously shown that in breast cancer, the direct and stable association of a tumor cell expressing Mena, a Tie2^hi^/VEGF^hi^ macrophage, and a vascular endothelial cell, creates an intravasation portal, called a “tumor microenvironment of metastasis” (TMEM) doorway, for tumor cell intravasation, leading to dissemination to distant sites. The density of TMEM doorways, also called TMEM doorway score, is a clinically validated prognostic marker of distant metastasis in breast cancer patients. Although we know that tumor cells utilize TMEM doorway-associated transient vascular openings to intravasate, the precise signaling mechanisms involved in TMEM doorway function are only partially understood. Using two mouse models of breast cancer and an in vitro assay of intravasation, we report that CSF-1 secreted by the TMEM doorway tumor cell stimulates local secretion of VEGF-A from the Tie2^hi^ TMEM doorway macrophage, leading to the dissociation of endothelial junctions between TMEM doorway-associated endothelial cells, supporting tumor cell intravasation. Acute blockade of CSF-1/CSF-1R signaling decreases macrophage VEGF-A secretion as well as TMEM doorway-associated vascular opening, tumor cell trans-endothelial migration, and dissemination. These new insights into signaling events regulating TMEM doorway function should be explored further as treatment strategies for metastatic disease.

## Introduction

Metastasis is a complex, multi-step and multi-directional process that begins with the dissemination of tumor cells from the primary tumor to distant sites and escalates by further re-dissemination of cancer cells from metastatic foci [1–4]. The re-dissemination process leads to an exponential increase in tumor burden and, eventually, patient demise. Tumor cell intravasation into the vasculature creates circulating tumor cells (CTCs) and is an essential step in the metastatic cascade, but its exact mechanism is not completely understood. A large body of evidence indicates that tumor cell dissemination, including tumor cell intravasation, is orchestrated by the interaction of tumor cells with the tumor microenvironment [5, 6]. In particular, tumor-associated macrophages interact with tumor cells and promote directed cell migration (streaming) towards blood vessels [7–9]. In addition, macrophages form a multi-cellular complex with endothelial and tumor cells on the surface of blood vessels to mediate transient vascular opening and tumor cell intravasation. These cell complexes are called TMEM (Tumor MicroEnvironment of Metastasis) doorways [10, 11]. Each TMEM doorway is composed of one tumor cell expressing Mena, one Tie2^hi^/VEGF-A^hi^ macrophage, and a vascular endothelial cell, all in direct and stable physical contact [10, 12, 13].

TMEM doorway-associated vascular opening (TAVO) was first discovered using high-resolution intravital imaging as localized and transient bursts of intravascular contrast agents into the extravascular space that could be accompanied by concurrent intravasation of cancer cells [10]. Further analyses in mouse models of breast cancer revealed that tumor cell intravasation occurs exclusively at TMEM doorways, following a TAVO event [10]. Importantly, the density of TMEM doorways, called TMEM doorway score, in primary breast tumors has been clinically validated as a prognostic marker of distant metastatic recurrence in breast cancer patients with estrogen receptor-positive HER2-negative (ER^+^/HER2^-^) disease, independent of other clinical prognostics [12–14]. Moreover, TMEM doorway score has been shown to increase in breast cancer patients following neo-adjuvant chemotherapy [15], highlighting the importance of understanding how the cells within TMEM doorways signal to activate the opening of TMEM doorway, promoting tumor cell dissemination.

In humans and mice, TMEM doorways are found in pre-invasive and invasive ductal breast carcinoma, as well as in metastatic foci in lymph nodes and lungs [16–22]. This suggests that the TMEM doorway-mediated mechanism of cancer cell dissemination occurs not only at the primary tumor site, but also at metastatic sites, and may contribute to the multi-directional cancer spread that perpetuates metastatic dissemination even after removal of the primary tumor [1–4, 16]. Thus, understanding the molecular mechanisms of TMEM doorway function may help develop therapeutic targets to slow distant metastases and improve patient survival.

Inhibiting TAVO prevents the formation of CTCs and metastases [10]. Although we know that TMEM doorways induce localized vascular opening through the secretion of vascular endothelial growth factor-A (VEGF-A) from the TMEM doorway macrophage [10], the precise signaling mechanisms involved in TMEM doorway function during intravasation have not been elucidated. The work described here provides a greater understanding of the signaling events that regulate these dynamic, multi-cellular interactions. In particular, we report the identification of the signaling events involved in TMEM doorway-mediated intravasation, which can serve as potential new prognostic markers and therapeutic targets for suppressing CTC dissemination and breast cancer metastasis.

## Materials and methods

### Cell culture

MDA-MB-231 cells were cultured in Dulbecco’s Modified Eagle Medium (DMEM) (cat# SH30243, Hyclone, GE Healthcare Life Sciences, Logan, UT, USA) supplemented with 10% fetal bovine serum (FBS) (cat# S11550, Atlanta Biologicals, Flowery Branch, GA, USA). BAC1.2F5 macrophages [23] and bone marrow derived macrophages (BMMs) were cultured in Minimum Essential Medium, Alpha (α-MEM) (cat# 15-012-CV, Corning, Tewksbury, MA, USA) supplemented with 10% FBS (cat# 100-106, Gemini Bio-Products, Sacramento, CA, USA) and 36 µg/mL human recombinant CSF-1 (3000 U/mL, a gift from Dr. E. Richard Stanley). Immortalized BMMs derived from *Csf1r*^*+/+*^ and *Csf1r*^−^^*/−*^ mice have been previously described and were cultured in α-MEM supplemented with 10% FBS and GM-CSF [24]. Human Umbilical Vein Endothelial Cells (HUVECs) were cultured in complete EGM-2 (cat# CC-3162, Lonza, Allendale, NJ, USA) and were not used beyond passage five for any experiments. THP-1 cells were cultured in RPMI 1640 (cat#11875093, Thermo Fisher, Waltham, MA, USA) supplemented with 10% FBS. THP-1 cells were differentiated into macrophages with 100 ng/mL PMA for 48 h followed by 48 h in RPMI 1640 prior to use in experiments.

### Mice

All studies involving mice were conducted in accordance with the National Institutes of Health regulations concerning the care and use of experimental animals. The procedures were approved by the Albert Einstein College of Medicine Institute for Animal Care and Use Committee. Transgenic mice expressing the Polyoma Middle T (PyMT) antigen and dendra2 fluorescent protein under the control of the mammary tumor virus promoter (MMTV), MMTV-iCre/CAGCAT-Dendra2/MMTV-PyMT [25] were bred in house. *SCID* mice (strain #001303) were purchased from The Jackson Laboratory (Bar Harbor, ME, USA).

### Pre-clinical in vivo TMEM doorway inhibition studies

Inhibitors were administered based on established doses of efficacy found in previous studies. In CSF-1R antibody blocking experiments, tumor pieces from *MMTV-PyMT/dendra2* mice were orthotopically transplanted into syngeneic FVB mice. Following about seven weeks of tumor growth, tumors grew to approximately 1.5 cm^3^, and animals were divided into two treatment groups. Animals were treated with 2.5 μg of anti-CSF-1R neutralizing antibody (cat# NBP1-43363, clone AFS98, Novus Biologicals, Centennial, CO, USA), or antibody isotype control (cat# 554682, IgG K isotype control, BD Pharmingen, Franklin Lakes, NJ, USA). Antibodies were administered by tail vein *i.v*. 4 h before termination of the experiment as previously described [26]. In CSF-1 blocking animal experiments, HT17 human PDX tumor chunks were orthotopically transplanted into *SCID* mice, as previously described [15, 27, 28]. Following about six weeks of tumor growth, tumors grew to approximately 1.5 cm^3^, animals were divided into two groups, and were treated with 5 μg/mL IgG isotype control or human CSF-1 neutralizing antibody (cat# AB-216-NA; cat#AB-108-C, R&D Systems, Minneapolis, MN, USA). Antibodies were administered by tail vein 24 h before the termination of the experiment. In both CSF-1R and CSF-1 blocking antibody experiments, mice were injected *i.v*. with 20 mg/mL 155 kDa TMR-dextran (cat# T1287, Sigma-Aldrich, Burlington, MA, USA) diluted in PBS, 1 h before the termination of the experiments. To collect circulating tumor cells (CTCs), mice were anesthetized with isoflurane, and blood was collected from the right ventricle via cardiac puncture with a heparinized syringe and incubated with RBC lysis buffer (cat# 00-4333-57, Invitrogen) for 5 min, and neutralized with PBS. Cells were spun at 300 × *g* for 10 min at 4 °C and pellets were suspended and cultured in DMEM/F12 (cat# 11320033, Gibco) supplemented with 20% FBS. Adherent tumor cells were quantified as CTCs at time of no tumor cell growth, as previously described [10, 29]. Fluorescent tumor cells expressing Dendra2, used in the *MMTV-PyMT* transplantation model, were quantified using fluorescence microscopy. The raw number of CTCs for each mouse was normalized to the volume of blood collected for each mouse. The number of CTCs in the antibody blocking groups was then set relative to the average number of CTCs in the isotype control blocking group for each individual experiment so that experiments could be combined. Primary breast tumors were collected at the time of sacrifice and fixed in either 10% formalin or 4% paraformaldehyde and used for subsequent tissue staining.

### in vitro TMEM doorway function inhibitors

For in vitro studies, GW2580, a small molecule inhibitor of CSF-1R (cat# G-5903, LC Laboratories, Woburn, MA, USA) was dissolved in DMSO and used at a concentration of 100 nM. Anti-mouse CSF-1R neutralizing antibody or isotype control (clone AFS98, Novus Biologicals) was used at a concentration of 50 ng/mL. Anti-human CSF-1R antibodies and IgG controls (cat# MAB3291; cat# MAB002, R&D Systems) were reconstituted at 0.5 mg/mL in PBS and were used at a concentration of 50 ng/mL in experiments.

### Immunofluorescence labeling of tumor vasculature and extravasation with 155 kDa dextran-TMR

Labeling flowing vasculature and sites of permeability was performed as previously described [10]. Briefly, to quantify extravasation, high molecular weight 155 kDa TMR-dextran (cat# T1287, Sigma-Aldrich, Burlington, MA, USA) diluted in PBS was administered by tail vein *i.v*. 1 h before the termination of the experiments. Anti-mouse CD31-biotin (cat# 13-0311-82, Thermo Fisher, Waltham, MA, USA) was administered by tail vein *i.v*. 10 min before the end of the experiment to label flowing blood vessels. At time of sacrifice tumors were removed and fixed for 48 h in 10% formalin in a volume ratio of tumor to formalin of 1:7 and made into paraffin blocks. Paraffin blocks of tumors were cut into 5 µm sections and immunofluorescence staining was performed. TMR-Dextran is visualized using rabbit anti-TMR (A-6397; Life Technologies, Carlsbad, CA, USA).

### Immunofluorescence staining of tissue

Tumor sections were dewaxed in xylene and rehydrated in alcohol followed by water. Antigen retrieval was performed with a citrate solution at pH 5.5 or EDTA pH 9.0 in a humidified chamber. The slides were washed with PBS-T (0.1% Tween-20) and blocked with a blocking solution (2% BSA, 10% FBS in PBS). One 5 μm section from each tumor was stained for hematoxylin and eosin (H&E) and one for TMEM doorway. TMEM doorway stain is a triple immuno-stain for predicting metastatic risk, in which three antibodies are applied sequentially and developed separately with different chromogens on a Dako Autostainer [13, 14, 30]. The following primary antibodies were used for immunostaining of TMEM doorways: anti-Mena (cat# NBP1-87914, Novus Biologicals), anti-endomucin (cat# sc-65495, Santa Cruz Biotechnology, Dallas, TX, USA), and anti-CD68 (cat# MCA1957, clone FA-11, Serotec, Kidlington, UK), or anti-Iba1 (cat# 019-19741, FUJIFILM Wako Chemicals, Richmond, VA). The sequential tissue sections were stained with different combinations of: anti-endomucin (cat# sc-65495, Santa Cruz Biotechnology), anti-TMR (to visualize TMR-dextran, cat# A-6397, Thermo Fisher Scientific), anti-ZO-1 (cat# MABT11, clone R40.76, Millipore Sigma) or anti-ZO-1 (cat# 402200, Invitrogen), anti-CD31 (cat# 77699; Cell Signaling Technology, Danvers, MA, USA), Red fluorochrome (635)-conjugated anti-Iba1 (cat# 013-26471, FUJIFILM Wako Chemicals), or anti-Iba1 (cat# 019-19741, FUJIFILM Wako Chemicals), anti-VEGF-A (cat# sc-152, clone A-20, Santa Cruz Biotechnology) or anti-VEGF-A (cat#512809, clone 2G11-2A05, Biolegend, San Diego, CA, USA) or anti-VEGF-A (cat# MA5-32038,Thermo Fisher), anti-CSF-1R (cat# sc-692, clone C-20, Santa Cruz Biotechnology), Alexa Fluor555-conjugated anti-CSF-1 (cat# bs-4910R-A555, Bioss Inc, Woburn, MA, USA), anti-CD68 (cat# MCA1957, clone FA-11, Serotec), or Alexa Fluor647-conjugated CD68 (cat#51-0689-42, eBioscience, San Diego, CA, USA), anti-F4/80 (cat# 70076, Cell Signaling), and anti-MRC1/CD206 (cat# AF2535, R&D Systems, Minneapolis, MN, USA). Sections were washed with PBS-T and the primary antibodies were detected with Alexa Fluor 488, 555 or 647 conjugated secondary antibodies targeting the primary antibody species (Invitrogen, Eugene, OR, USA), and nuclei were stained with 4, 6-diamidino-2-phenylindole (DAPI). All fluorescently labeled samples were mounted with Prolong Diamond antifade reagent (cat# P36961, Invitrogen) and imaged with a PerkinElmer Pannoramic 250 Flash II digital whole-slide scanner using a 20 × 0.8NA Plan-Apochromat objective (PerkinElmer, Hopkinton, MA, USA). Images of individual fields of view were imported into ImageJ or VisioPharm for analysis.

### Extravascular dextran, VEGF-A, and intracellular CSF-1 quantification in TMEM doorways

To identify active TMEM doorways, we utilized an improved and automated method of a prior TMEM activity assay that takes into account the presence of high-molecular-weight dextran that leaks into the tissue, as a result of TMEM doorway-associated vascular opening (TAVO) [10, 15, 31]. The algorithmic improvement of this assay involves the automated measurement of the entire tissue section of the tumor as a single region of interest (ROI), instead of selecting a definite number of high power fields as ROIs, thus providing a more accurate representation of the entire tumor, minimizing operator-dependent biases, and significantly increasing the logistic capacity of the analysis.

In short, serial tissue sections were cut and stained for TMR-Dextran, CSF-1, and endomucin by IF (tissue section 1) and for TMEM doorways by staining for Iba1, endomucin, and Mena by IHC (tissue section 2). The sections were then imaged with a digital whole slide scanner and aligned to the single cell level using the TissueAlign module in Vis (Visiopharm, Hoersholm, Denmark). IF images were thresholded above background, creating binary masks for endomucin (blood vessel) and dextran signals. These masks were then superimposed so as to be able to differentiate between intravascular and extravascular dextran signal. In the IHC section, TMEM doorways were identified using previously published criteria [13, 14]. In this manner, the amount of extravascular dextran around TMEM doorways could be quantified. For quantification of intracellular CSF-1, the TMEM doorway-associated tumor cell was identified using the Mena stain in IHC section, within the TMEM doorway circle, and a mask was created. This mask was then overlaid onto the aligned IF section and the CSF-1 was then quantified within this mask.

### siRNA knockdown

MDA-MB-231 cells were transfected with Stealth RNAi siRNAs (siRNA IDs: HSS102355, HSS102356, HSS175321, Thermo Fisher Scientific) directed against CSF-1 or negative control (cat#12935300, Thermo Fisher Scientific) at a concentration of 200 pmols using Lipofectamine 2000 reagent (cat# 11668027, Invitrogen).

### Immunofluorescence staining of co-cultured cells

Prior to the start of the experiment, macrophages were labeled with CellTracker Green^TM^ dye and tumor cells were labelled with CellTracker^TM^ Red dye, according to manufacturer’s instructions (cat# C7025, C34552, Invitrogen). 1 × 10^5^ macrophages were cultured with or without 1 × 10^5^ tumor cells in α-MEM media (supplemented with 0.5% FBS and 300 U/mL of CSF-1 (one-tenth the amount of CSF-1 added to complete culture media)) and with either CSF-1R blocking antibody or isotype control antibody at 50 ng/mL (cat# NBP1-43363, clone AFS98, Novus Biologicals; IgG K isotype control cat# 554682, BD Pharmingen). After 24 h incubation, the experiments were stopped by washing the cells with ice-cold PBS followed by fixing them for 20 min in 4% PFA, and permeablizing in 0.1% Triton X-100 in PBS. The cells were stained for VEGF-A using antibodies against mouse VEGF-A (cat# 512809, clone 2G11-2A05, Biolegend) and Alexa Fluor647-conjugated secondary antibodies. The intensity of VEGF-A staining in the macrophages was measured by first outlining the macrophages in the green (488 nm) channel in ImageJ/Fiji [32] and applying that outline to the far-red (647 nm) channel to measure the VEGF-A signal within the macrophage, excluding any VEGF-A signal from the tumor cells.

### Quantitative real-time polymerase chain reaction (qPCR)

To quantify gene expression in MDA-MB-231 tumor cells transfected with CSF-1 siRNA or control siRNA, tumor cells were grown under normal culture conditions. In co-culture experiments between tumor cells and macrophages, 1 × 10^6^ macrophages were cultured alone or co-cultured with 1 × 10^6^ MDA-MB-231 tumor cells for 24 h in α-MEM media with 0.5% FBS and 300 U/mL of CSF-1 (one-tenth of CSF-1 added to complete media). For the co-culture assay, species-specific primers were used in the qPCR.

Total RNA was isolated from cells by using the RNeasy Plus Mini Kit (cat# 74134, Qiagen, Germantown, MD, USA) and cDNA was synthesized and amplified from 1 μg total RNA using the using SuperScript IV VILO Master Mix with ezDNase Enzyme (cat# 11766050, Thermo Fisher Scientific) per manufacturer’s protocol. The qPCR was performed with SYBR Green PCR Master Mix (cat# 4367659, Thermo Fisher Scientific) using a QuantStudio 3 real-time PCR instrument (applied biosystems, Thermo Fisher Scientific). The following primers were used: mouse GAPDH 5’-CTCATGACCACAGTCCATGC-3’, 5’-CACATTGGGGGTAGGAACAC-3’; mouse VEGF-A 5’-AGCAGAAGTCCCATGAAGTGA-3’, 5’-ATGTCCACCAGGGTCTCAAT-3’; human GAPDH 5’-CTCCTGTTCGACAGTCAGCC-3’, 5’-ACCAAATCCGTTGACTCCGAC-3’; human β-Actin 5’-CTTCGCGGGCGACGATGC-3’, 5’-CGTACATGGCTGGGGTGTTG-3’; human CSF-1 5’-CCTCCCACGACATGGCT-3’, 5’-GAGACTGCAGGTGTCCACTC-3’. The mean cycle threshold (Ct) values were then used to analyze relative expression. Analysis was performed using comparative-CT method (2^−ΔΔCT^ method) and all Ct values were normalized to GAPDH. Each reaction was performed in triplicate.

### ELISA

In macrophage-tumor cell co-culture experiments, 1 × 10^6^ macrophages were cultured alone or co-cultured with 1 × 10^6^ MDA-MB-231 tumor cells for up to 48 h in α-MEM media with 0.5% FBS and 300 U/mL of CSF-1 (one-tenth of CSF-1 added to complete media). Experiments using immortalized *Csf1r*^*+/+*^ or *Csf1r*^*−/−*^ BMMs used α-MEM media with 0.5% FBS and 2 ng/mL GM-CSF. Prior to co-culturing with MDA-MB-231 tumor cells or tumor cell conditioned media, human THP-1 monocytes were differentiated into macrophages by adding PMA at 100 ng/mL to the culture media for 48 h. In mono-culture macrophage ELISA experiments where tumor cell conditioned media was added, conditioned media was collected from tumor cells incubated in α-MEM with 0.5% FBS and 300 U/mL of CSF-1 for 24 h. In tumor cell mono-culture ELISA experiments, 1 × 10^6^ tumor cells were seeded and incubated for 24 h in DMEM with 0.5% FBS.

In all ELISA experiments, blocking antibodies, isotype controls, inhibitors and controls were added at indicated concentrations in “in vitro inhibitors” section, at time zero. Media conditioned by the cultured cells were collected at the indicated time points and stored at -80 ^°^C until use. ELISAs were performed per the manufacturer’s recommendations using the Mouse VEGF-A DuoSet ELISA kit (cat# DY493, R&D Systems), the Human M-CSF DuoSet ELISA kit (cat# DY216, R&D Systems), or the Human VEGF-A DuoSet ELISA kit (cat#DY293B-05). The concentration of protein secreted was interpolated from the standard curve measurements.

### Trans-endothelial Migration Assay (iTEM assay)

The iTEM assay was performed as previously described and briefly described here. The transwell (8 µm pore size; cat# 353097, Corning, Corning, NY, USA) was prepared so that tumor cell trans-endothelial migration was in the intravasation direction found in vivo (from subluminal side to luminal side of the endothelium). To prepare the endothelial monolayer, the underside of each transwell was coated with 50 µL of Matrigel (2.5 µg/mL; cat# 354230, Corning). Approximately 1 × 10^5^ HUVECs were plated on the Matrigel-coated underside of the transwell. Transwells were then flipped into a 24-well plate containing 200 µL of complete EGM-2 and monolayers were formed over a 48 h period. The integrity of the endothelium was measured using low molecular weight dextran as described previously [33]. Macrophages and tumor cells were labelled with cell tracker dyes (cat# C7025, C34552, Invitrogen) before the experiment. Then, 1.5 × 10^4^ tumor cells and 6 × 10^4^ macrophages were added to the upper chamber of the transwell in 200 µL of DMEM without serum while the bottom chamber contained EGM-2 supplemented with 36 µg/mL of CSF-1. After 18 h, the transwells were fixed and stained for ZO-1 (cat# 402200, Invitrogen) as previously described. Transwells were imaged using a Leica SP5 confocal microscope using a 60 × 1.4 NA objective and processed using ImageJ/Fiji [32]. Tumor cell trans-endothelial migration quantification was performed by counting the number of tumor cells that had crossed the intact endothelium (intact monolayers confirmed by ZO-1 staining for tight junctions) within the same field of view (60×, 10 random fields) and represented as normalized values from at least three independent experiments.

### Statistical analysis

Individual animals in each cohort are presented as individual points on a dot plot. A horizontal line indicates the mean value and the error bars represent the standard deviation or standard error of the mean, as indicated in the figure legend. Statistical significance was determined using an unpaired Student’s *t*-test, one-way ANOVA, or two-way ANOVA, with Tukey’s multiple comparisons test, as indicated, using GraphPad Prism (version 10; Graph Pad Software, La Jolla, CA). Correlation was determined using Pearson’s Correlation Coefficient. Data sets were checked for normality (D’Agostino & Pearson omnibus normality test or Shapiro-Wilk normality test) and unequal variance using GraphPad Prism. Welch’s correction was applied to *t*-tests as needed. *P* values less than 0.05 were deemed significant. For in vitro experiments, results are representative of at least three independent experiments. Power analyses were performed to determine the sample size needed to obtain statistical significance for all experiments. The ROUT method (GraphPad Prism, version 10) was used to identify outliers from nonlinear regression.

## Results

### CSF-1 levels are elevated in tumor cells at active TMEM doorways

**Fig. 1:**
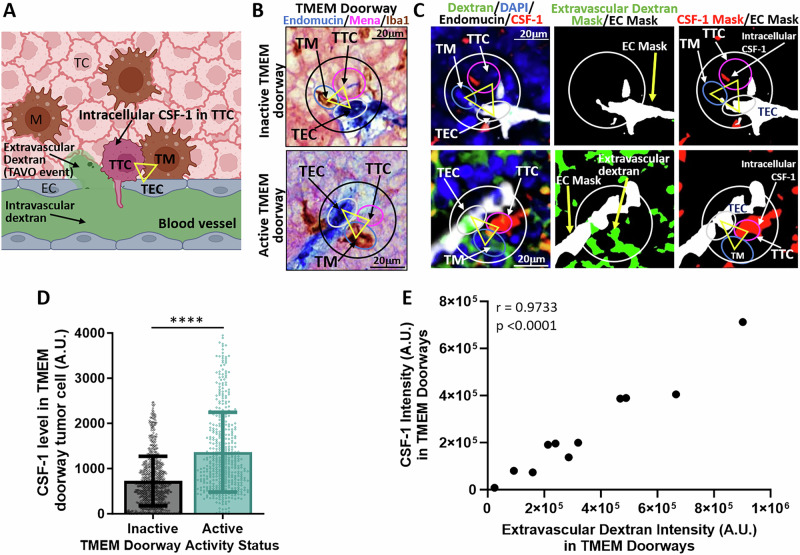
TMEM doorway tumor cells show increased CSF-1 levels at active TMEM doorways. **A** Cartoon denoting the identification of parameters used for analysis of CSF-1 levels in TMEM doorway tumor cells (TTCs) and TMEM doorway function. The three cells composing the TMEM doorway are indicated by the yellow triangle connecting the TMEM doorway macrophage (TM), the TMEM doorway endothelial cell (TEC) and the TTC. Intravascular dextran (green) is in the lumen of blood vessel, extravascular dextran is outside the lumen of blood vessel, and CSF-1 is directly measured in the TTC. Figure created with BioRender.com. **B** Panel shows inactive and active TMEM doorways, visualized by immunohistochemistry (IHC) staining for Mena, Iba-1, and endomucin. The three cells of the TMEM doorway (contained in black circle, with the three cells forming the TMEM doorway indicated with the yellow triangle) are the TMEM doorway endothelial cell (TEC, endomucin stained in blue, circled in white), TMEM doorway macrophage (TM, Iba1 stained in brown, circled in teal), and TMEM doorway tumor cell (TTC, Mena stained in pink, and circled in pink) and black arrows indicate where the cells are localized within the TMEM doorway. TMEM doorways were identified using automated analysis by VisioPharm identifying three adjacent immuno-histochemical stains. **C** The sequential tissue sections after the IHC in (**B**) were stained using immunofluorescence (IF) with antibodies against endomucin (white), dextran (green), CSF-1 (red), and nuclear stain DAPI (blue). The two sequential sections from **B**,**C** were aligned and the same TMEM doorways were matched between the two sections, as indicated by the black circle in the IHC panel (**B**) and white circle in the IF panels (**C**). The left panels demonstrate the association of CSF-1 level in the TMEM doorway tumor cell (TTC) with TMEM doorway activity. The middle panel shows the extravascular signal for dextran as a green mask and the endomucin stain as a white mask, where thresholded, positive signal for these stains was converted into a binary mask. Active versus inactive TMEM doorways were distinguished by the presence of extravascular dextran staining (non-overlapping with the endomucin stain), which indicates that the vessel had a TMEM doorway-associated vascular opening (TAVO). Scale bars = 20 μm. **D** Immunofluorescence measurement of CSF-1 levels in TMEM doorway tumor cells in active and inactive TMEM doorways. Active and inactive TMEM doorways were identified in the IF-stained sections as described above (see **C**). Next, tumor cells were identified using the Mena-positive cells at TMEM doorway (**B**, IHC stain). The level of CSF-1 in the TMEM doorway tumor cells was measured in both active and inactive TMEM doorways using Visiopharm. Between 82 and 134 TMEM doorways were analyzed per mouse, in 12 mice. Each point represents one TMEM doorway, bars show the mean intensity (AU) and error bars represent standard deviation. ****P < 0.0001 analyzed by Student’s *t*-test. **E** Average immunofluorescence intensity plot of CSF-1 staining within TMEM doorways compared to the average intensity of extravascular dextran within TMEM doorways in tumor tissues stained and aligned as in Fig. 1B, C. *n* = 12 mice, each point represents the average value per mouse. Pearson correlation coefficient *r* = 0.9733, *p* < 0.0001.

Macrophage-secreted VEGF-A is critical for the regulation of TMEM doorway activity [10] and VEGF-A expression in macrophages has been shown to be regulated by CSF-1, also known as M-CSF [34, 35]. Since tumor cells are known to secrete CSF-1, we investigated if TMEM doorway tumor cells express CSF-1, and if this expression is associated with TMEM doorway vascular opening [10]. We first determined whether CSF-1 levels are increased in TMEM doorway tumor cells at active TMEM doorways by multiplex staining of breast tumor tissue sections obtained from the *polyoma* middle T antigen (PyMT) mice injected *i.v*. with high molecular weight dextran (155 kDa, green) 1 h before sacrifice. In these samples, if there has been a TMEM doorway-associated vascular opening (TAVO) (which would indicate an active TMEM doorway), the *i.v*. injected dextran would extravasate into the interstitium and be detected extravascularly in the tissue near a TMEM doorway **(**Fig. 1A**)**. We used a modified approach of our previously published protocol for detection of active TMEM doorways [31]. We stained two sequential tumor sections – one using IHC for TMEM doorways (Iba1, Endomucin, Mena) (Fig. 1B) and the second using IF for CSF-1, dextran, and endothelial cells (endomucin) (Fig. 1C) and aligned the images of stained sections to the single cell level. Next, we identified which TMEM doorways were active by examining for the presence of high molecular weight dextran (155 kDa, green) in the extravascular space near the TMEM doorway, as described previously [10, 15]. We then visualized and quantified the expression of CSF-1 (red) in TMEM doorway tumor cells of both active and inactive doorways **(**Fig. 1C**)**. We observed that the fluorescence intensity of intracellular CSF-1 in TMEM doorway tumor cells was higher in active TMEM doorways (Fig. 1C, lower panel) compared to inactive TMEM doorways (Fig. 1C, upper panel). This is quantified in Fig. 1D. We further compared the intensity of CSF-1 staining within TMEM doorways to the intensity of extravascular dextran staining within TMEM doorways and found a significant correlation between these two measurements, with an r value of 0.9733 (Fig. 1E). These results indicate that TMEM doorway tumor cells may play a significant role in regulating TMEM doorway function and activity by locally producing CSF-1.

### Tumor cells stimulate macrophage VEGF-A secretion without affecting its expression

**Fig. 2:**
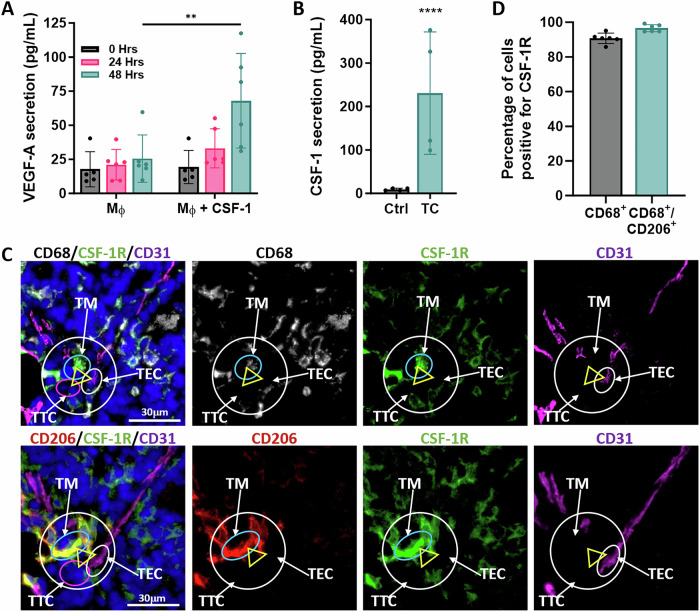
TMEM doorway macrophages secrete VEGF-A in response to CSF-1 and tumor cells secrete CSF-1. **A** VEGF-A ELISA of conditioned media obtained from BAC1.2F5 macrophages (Mϕ) treated with or without CSF-1 (3000 U/mL) for the times indicated. VEGF-A is indicated as concentration (pg/ml) of secreted protein. *n* = 3 individual experiments performed in duplicate, **p < 0.01, by two-way ANOVA. **B** CSF-1 ELISA of conditioned media obtained from MDA-MB-231 tumor cells (TCs). TCs were cultured for 24 h in serum-free media and the concentration of CSF-1 (pg/mL) secreted by the TCs measured in the tumor cell conditioned media by ELISA. Control is media not exposed to tumor cells but treated in the same way as the cells. *n* = 3 individual experiments performed in duplicate. ****p < 0.0001 by Student’s *t-*test. **C** Immunofluorescence staining of serial PyMT tumor sections stained for CSF-1R (green), vasculature (CD31, magenta), macrophages (top: CD68, white; bottom: CD206, red) and DAPI (blue). The TMEM doorway is indicated with the yellow triangle, with each point of the triangle identifying each cell in the doorway, as described in Fig. 1 [10]. TMEM doorway cells are indicated with white arrows: endothelial cell (TEC, white circle); TMEM doorway macrophage (TM, blue circle); and TMEM doorway tumor cell (TTC, pink circle). Scale bar = 30 µm. **D** Quantification of the percent of CD68^+^ TMEM doorway macrophages and CD68^+^/CD206^+^ macrophages which stain positively for CSF-1R (*n* = 6 mice, each dot represents the average value per mouse).

Since CSF-1 is upregulated in the tumor cell at active TMEM doorways, and secretion of VEGF-A is known to be regulated by CSF-1 in other cell types, we investigated the signaling interplay between the tumor cell and macrophage at the TMEM doorway. Using an ELISA on conditioned media, we confirmed that exogenous recombinant CSF-1 can stimulate macrophages to secrete VEGF-A (Fig. 2A) and that tumor cells secrete CSF-1 (Fig. 2B). To examine the role of tumor cell secreted CSF-1 in regulating macrophage VEGF-A secretion, we first determined if TMEM doorway macrophages express the CSF-1 receptor (CSF-1R). We have previously identified TMEM doorway macrophages by their direct contact with a tumor cell and blood vessel [13], expression of CD68, Tie2, and VEGF-A, and further characterized them as a CD206^+^/CD11b^+^/F4/80^+^/CD11c^−^ population [10]. Using immunofluorescence staining of PyMT tumors for CSF-1R, macrophages (CD68^+^/CD206^+^/CD31^−^), and endothelial cells (CD31^+^) as described previously, we found that greater than 90% of all CD68^+^ macrophages throughout the tumor tissue co-express CSF-1R and 97% of CD68^+^/CD206^+^ TMEM doorway macrophages (identified by simultaneous contact with tumor cells and endothelial cells and co-expression of CD68 and CD206 without CD31 expression) also express CSF-1R (Fig. 2C, D).

**Fig. 3:**
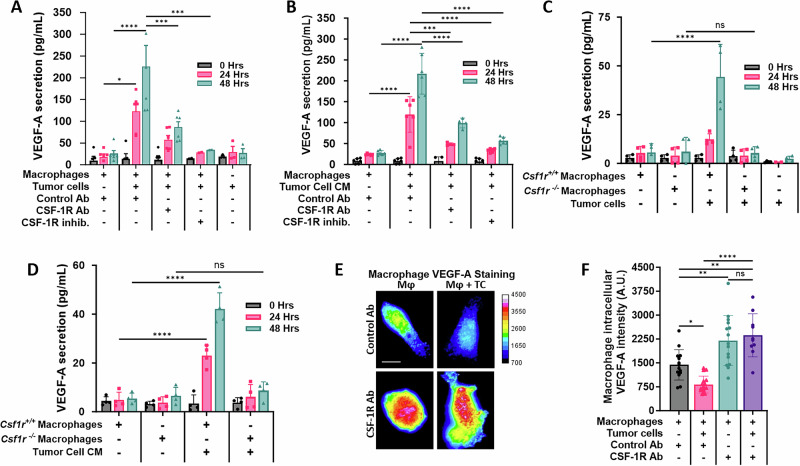
Tumor cell secreted CSF-1 increases CSF-1R-dependent macrophage VEGF-A secretion. **A** VEGF-A ELISA of conditioned media obtained from BAC1.2F5 macrophages co-cultured with MDA-MB-231 tumor cells treated with control antibody (Control Ab), CSF-1R blocking Ab or CSF-1R inhibitor (GW2580), denoted as concentration (pg/mL) of secreted protein. *n* = 3 individual experiments performed in duplicate, *p < 0.05, ***p < 0.001, ****p < 0.0001 analyzed by two-way ANOVA. **B** ELISA determination of VEGF-A (pg/mL) in medium obtained from BAC1.2F5 macrophages cultured in medium conditioned by MDA-MB-231 tumor cells (tumor cell CM) and treated with control Ab, CSF-1R blocking Ab (CSF-1R Ab) or CSF-1R inhibitor (GW2580). *n* = 3 individual experiments performed in duplicate, ***p < 0.001, ****p < 0.0001 analyzed by two-way ANOVA. **C** ELISA determination of VEGF-A (pg/mL) in medium conditioned by bone marrow macrophages (BMMs) either expressing (*Csf1r*^*+/+*^) or lacking (*Csf1r*^−^^*/−*^*)* CSF-1R co-cultured with MDA-MB-231 tumor cells for the times indicated. *n* = 3 individual experiments done in duplicate, ns= not significant, ****p < 0.0001 analyzed by two-way ANOVA. **D** ELISA determination of VEGF-A (pg/mL) in medium from *Csf1r*^*+/+*^ or *Csf1r*^*−/−*^ BMMs cultured in MDA-MB-231 tumor cell conditioned medium (tumor cell CM) for the times indicated. *n* = 3 individual experiments performed in duplicate, ns= not significant, ****p < 0.0001 analyzed by two-way ANOVA. **E** BAC1.2F5 macrophages (Mφ), labelled with CellTracker^TM^ Green, were co-cultured with or without MDA-MB-231 tumor cells (TC), labelled with CellTracker^TM^ Red, and treated with either control or CSF-1R blocking antibodies for 48 h. Cells were fixed, permeabilized and stained for VEGF-A. Macrophages were identified by the CellTracker Green label and only images of macrophages are shown. The heat map scale to right shows intensity of VEGF-A staining. Scale bar = 5 µm. **F** Quantitation of the VEGF-A fluorescence intensity in macrophages in (E). The amount of VEGF-A in the macrophage was quantified using ImageJ, as described in the methods section. ns not significant, *p < 0.05, **p < 0.01, ****p < 0.0001, analyzed by one-way ANOVA. Experiment performed in triplicate, one representative experiment shown with at least 10 cells analyzed per group, each dot represents a cell.

To determine if tumor cells can stimulate macrophage VEGF-A secretion, we used a human tumor cell–mouse macrophage xenogenic co-culture system and quantified the amount of secreted VEGF-A by ELISA using an antibody that can only recognize mouse-derived VEGF-A, secreted by the macrophages. Co-culture of human MDA-MB-231 tumor cells with mouse BAC1.2F5 macrophages (Fig. 3A) or culture of BAC1.2F5 macrophages with conditioned media collected from serum-starved MDA-MB-231 tumor cells (Fig. 3B), resulted in increased secretion of VEGF-A by the mouse-derived macrophages.

To ascertain the role of tumor cell-secreted CSF-1 in stimulating macrophage VEGF-A secretion, we used the same co-culture system of MDA-MB-231 tumor cells and BAC1.2F5 macrophages as above. We inhibited CSF-1R signaling in macrophages with either a CSF-1R neutralizing antibody (CSF-1R Ab), or an inhibitor which prevents downstream signaling of the CSF-1R (CSF-1R inhib. (GW2580)). We found that either method reduced macrophage VEGF-A secretion (Fig. 3A). Likewise, inhibition of CSF-1R in macrophages cultured with medium conditioned by the tumor cells led to a reduction in the amount of secreted VEGF-A (Fig. 3B). Similar results were obtained using mouse bone marrow derived primary macrophages (BMMs) (Supplementary Fig. 1A). Co-culturing a different breast cancer cell line, 4T1, which also secretes CSF-1 (Supplementary Fig. 1B), with BAC1.2F5 macrophages also caused an increase in VEGF-A secretion by macrophages (Supplementary Fig. 1C). To control for any differences in signaling between species in our xenogenic assay, we also co-cultured the human-derived monocyte THP-1 cell line, after differentiating into macrophages, with human MDA-MB-231 tumor cells or conditioned media, and found the tumor cells and conditioned media increased the secretion of VEGF-A by the THP-1 cells (Supplementary Fig. 1E–G). In these additional models, the increase in macrophage secretion of VEGF-A in response to tumor cell CSF-1 was again suppressed through addition of CSF-1R inhibitors or blocking antibodies (Supplementary Fig. 1A, C, F, G). Next, we used immortalized bone marrow macrophages (BMMs) isolated from *Csf1r*^*+/+*^ or *Csf1r*^*−/−*^ mice which either express (*Csf1r*^*+/+*^) or lack the expression of CSF-1R (C*sf1r*^*−/−*^*)* to determine the role of the CSF-1R in regulating VEGF-A secretion by macrophages. Compared to the *Csf1r*^*+/+*^ BMMs, which secreted VEGF-A in both the co-culture system and when cultured in tumor cell conditioned media (Fig. 3C, D), C*sf1r*^*−/−*^ BMMs did not significantly secrete VEGF-A, in either a co-culture system with MDA-MB-231 tumor cells, or when cultured with medium conditioned by the tumor cells (Fig. 3C, D).

We next evaluated if co-culture of tumor cells with macrophages leads to increased expression or secretion of VEGF-A from macrophages, or both. We co-cultured MDA-MB-231 tumor cells with BAC1.2F5 macrophages labeled with CellTracker^TM^ Green to distinguish them from cancer cells, and then stained the cells for VEGF-A. Macrophages that were co-cultured with tumor cells displayed decreased intracellular VEGF-A staining compared to macrophages cultured alone (Fig. 3E, F). This result, combined with the VEGF-A ELISA showing that macrophages and tumor cells cultured in the same method led to increased VEGF-A in the conditioned media (Fig. 3A), suggests that the tumor cells induce macrophages to secrete VEGF-A. In addition, inhibition of CSF-1R signaling (which prevented VEGF-A secretion in Fig. 3A) increased intracellular macrophage VEGF-A levels using the same co-culture system (Fig. 3E, F), indicating that the CSF-1/CSF-1R signaling between macrophages and tumor cells induces the release of intracellular VEGF-A from macrophages. In our second model using 4T1 tumor cells, we again found a decrease in intracellular VEGF-A staining in the macrophages co-cultured with tumor cells compared to macrophages cultured without tumor cells (Supplementary Fig. 1D). The increase in macrophage VEGF-A secretion and the decrease in the intracellular VEGF-A protein levels in the macrophages were abrogated with CSF-1R neutralizing antibody as well as with the small molecule CSF-1R inhibitor, consistent to what was observed with MDA-MB-231 cells in Fig. 3.

To determine if the enhancement of VEGF-A secreted by the macrophages in response to co-culture with tumor cells could also be due to increased VEGF-A mRNA production, we co-cultured MDA-MB-231 tumor cells with BAC1.2F5 macrophages and determined the relative levels of VEGF-A mRNA by qPCR. We found that co-culture of tumor cells and macrophages did not affect the VEGF-A mRNA levels in macrophages (Supplementary Fig. 2A), indicating that the increase in VEGF-A protein found in the conditioned medium was due to increased secretion of stored intracellular VEGF-A in the macrophages, not also caused by an increase in VEGF-A mRNA expression.

### Inhibition of CSF-1R reduces TMEM doorway-associated vascular opening and trans-endothelial migration

**Fig. 4:**
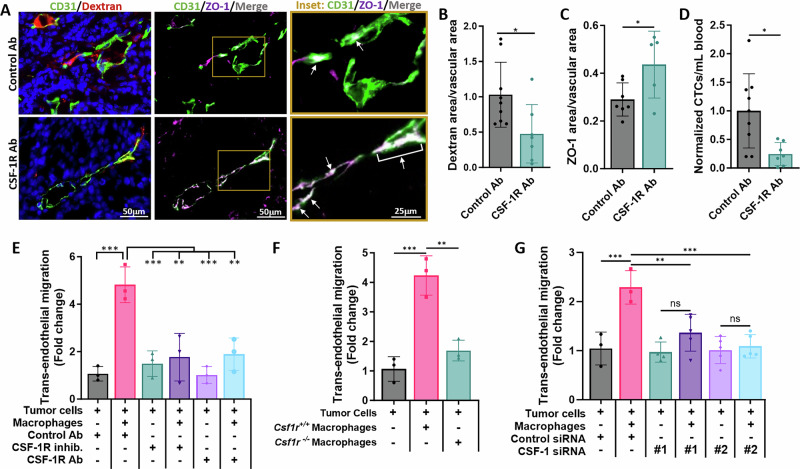
Inhibition of CSF-1R signaling reduces TAVO and trans-endothelial migration. **A** IF staining of tumor tissue from mice treated with control or CSF-1R blocking antibodies (CSF-1R Ab) were stained for CD31 (green), TMR-dextran (red), and ZO-1 (magenta). The right panel shows increased magnification of yellow box from middle panel to demonstrate overlap between CD31 and ZO-1 stains (merge/overlap indicated with white signal and indicated with white arrows). Scale bar left and middle panels= 50 µm; right panel= 25 µm, **B** Quantification of extravascular 155 kDa dextran-TMR. *p < 0.05. **C** Vascular ZO-1 *p < 0.05, and **D** circulating tumor cells for mice treated with control Ab or CSF-1R blocking Ab in **A**. *p < 0.05, analyzed by Student’s *t*-test. (**B**–**D**, *n* = 9 mice trea*t*ed with control antibody, 7 mice treated with CSF-1R antibody). **E** Quantitation of subluminal to luminal trans-endothelial migration (iTEM activity, which is a measure of number of intravasating tumor cells in the intravasation direction) of MDA-MB-231 cells plated on the confluent and sealed endothelium either alone or with BAC1.2F5 macrophages. Tumor cells were treated with control antibody (Control Ab and DMSO), CSF-1R blocking Ab (CSF-1R Ab) or CSF-1R inhibitor (GW2580). Tumor cells were labelled with CellTracker^TM^ green, which allowed us to identify and quantify the cells crossing the endothelial monolayer. The endothelium is stained with ZO-1 antibodies to ensure endothelial confluence and tight junctions at locations of TC crossing. The number of MDA-MB-231 cells that crossed the endothelium were imaged using a confocal microscope and quantified using ImageJ software. *n* = 3 experiments, each point represents the average fold change for that treatment group for each experiment, *p < 0.05 analyzed by one-way ANOVA. **F** Quantitation of iTEM activity of MDA-MB-231 cells plated on the endothelium alone or with BMMs expressing or lacking CSF-1R expression. The assay was imaged and analyzed as in (**E**). *n* = 3 experiments, each point represents the average fold change for that treatment group for each experiment, ***p < 0.001 analyzed by one-way ANOVA. **G** Quantitation of iTEM activity of MDA-MB-231 cells transfected with either control siRNA or two different siRNAs targeting CSF-1 (#1, #2). Transfected cells were plated on the endothelium either alone or with BAC1.2F5 macrophages. The assay was imaged and analyzed as in (**E**). *n* = 3 experiments, each point represents the average fold change for that treatment group for each experiment, ns not significant, **p < 0.01, ***p < 0.001 analyzed by one-way ANOVA.

As CSF-1R signaling regulates macrophage VEGF-A secretion, and macrophage-secreted VEGF-A is required for vascular opening at TMEM doorways [10], we examined the role of the CSF-1R in mediating vascular opening and tumor cell intravasation at TMEM doorways in vivo, as defined in Fig. 1 and previously [6]. We injected tumor-bearing PyMT mice *i.v*. with CSF-1R blocking antibodies or IgG control antibodies, 4 h prior to sacrifice, and injected high molecular weight dextran (155 kDa), 1 h before sacrifice (experimental model diagramed in Supplementary Fig. 2B). We stained the tumor tissue for CD31, TMR-Dextran, and ZO-1 to examine the effects of CSF-1R inhibition on TMEM doorway activity and vascular junctional integrity (Fig. 4A). Acute inhibition of CSF-1R reduced extravascular dextran (red) at TMEM doorways, indicating that TMEM doorways were less active when CSF-1 signaling is inhibited (Fig. 4B). Colocalization of vascular ZO-1, a component of endothelial tight junctions that maintains vascular junction integrity, with the adherens junction protein, CD31, increased with inhibition of CSF-1R suggesting that the endothelial junctions were more stable upon CSF-1R inhibition (Fig. 4C). To determine whether blockade of CSF-1R signaling and subsequent inhibition of TMEM doorway opening prevented tumor cells from entering the vasculature, we collected the blood from the mice during sacrifice and measured the number of circulating tumor cells (CTCs). The number of CTCs were significantly reduced upon CSF-1R inhibition compared to control treated mice (Fig. 4D). These results indicate that CSF-1R signaling is not only involved in tumor cell and macrophage streaming migration, as reported previously [8], but also in signaling between tumor cells, macrophages, and endothelial cells, creating intravasation-associated vascular openings and allowing intravasation of tumor cells, leading to CTC formation.

To further examine the effects of blocking CSF-1R signaling on macrophage-mediated trans-endothelial migration of tumor cells, we used our previously established in vitro trans-endothelial migration (iTEM) assay [36]. The iTEM assay mimics the conditions seen at the TMEM doorway during intravasation and allows us to quantify the number of tumor cells that cross the endothelium. We have previously shown that breast tumor cells seeded alone atop a Matrigel matrix and confluent endothelial monolayer cross through the endothelial cell barrier poorly, but trans-endothelial migration is enhanced in the presence of macrophages [36, 37]. As shown before, tumor cells exhibited a basal level of trans-endothelial migration, which was significantly increased in the presence of macrophages (Fig. 4E). However, macrophage-mediated trans-endothelial migration of tumor cells was decreased in the presence of either a CSF-1R blocking antibody or a small molecule inhibitor of CSF-1R (Fig. 4E).

To further establish that CSF-1R signaling between tumor cells and macrophages was important for increasing tumor cell migration across the endothelial cell layer, we used BMMs lacking CSF-1R (*Csf1r*^*−/−*^) in the iTEM assay. We observed no increase in the transmigration of tumor cells seeded with *Csf1r*^*−/−*^ macrophages compared to tumor cells cultured without macrophages; however, tumor cell trans-endothelial migration was enhanced when seeded with macrophages expressing the CSF-1R (*Csf1r*^*+/+*^) (Fig. 4F). Similarly, to establish that CSF-1 secreted by tumor cells specifically is responsible for the increased trans-endothelial migration, and not the other TMEM doorway cells present in the iTEM assay, we knocked down CSF-1 in MDA-MB-231 cells using siRNA (Supplementary Fig. 3A) and used these tumor cells in the iTEM assay. We found that unlike tumor cells expressing CSF-1, tumor cells with reduced CSF-1 expression did not support increased trans-endothelial migration in the presence of macrophages (Fig. 4G). Thus, inhibition of CSF-1R signaling blocked the ability of macrophages to enhance tumor cell trans-endothelial migration.

### Inhibition of tumor cell-derived CSF-1 in vivo blocks TMEM doorway-associated vascular opening and trans-endothelial migration

**Fig. 5:**
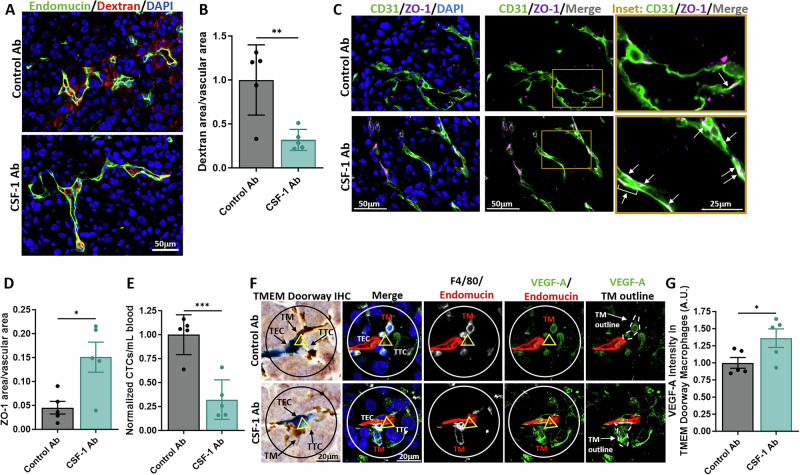
Specific inhibition of tumor-derived CSF-1 signaling in vivo reduces TAVO, trans-endothelial migration, and VEGF-A secretion in TMEM doorway macrophages. **A** IF staining of tumor tissue from mice treated with control or CSF-1 blocking antibodies (CSF-1 Ab) were stained for endomucin (green), TMR-dextran (red), and DAPI (blue). Scale bar = 50 µm. **B** Quantification of extravascular 155 kDa TMR-dextran. **p < 0.01, analyzed by Student’s *t*-test, *n* = 5 mice per group, each dot represents the average value for a mouse. **C** Mice from (**A**) stained with CD31 (green), ZO-1 (magenta), and DAPI (blue). The right panel shows increased magnification of the yellow box from middle panel to demonstrate overlap between CD31 and ZO-1 stains (merge/overlap indicated with white signal and indicated with white arrows). Left and middle panels scale bar = 50 µm; left panel = 25 µm. **D** Quantification of vascular ZO-1 stains in (**C**), *p < 0.05 analyzed by Student’s *t*-test, *n* = 5 mice per group, each dot represents the average value for a mouse. **E** Quantification of circulating tumor cells for mice treated with control Ab or CSF-1 blocking Ab in (**A**). ***p < 0.001, analyzed by Student’s *t*-test, *n* = 5 mice per group, each dot represents the normalized value for a mouse. **F** Immunostaining of VEGF-A intensity in TMEM doorway macrophages, obtained from mice in (**A**). Sequential tumor sections were stained by IHC (TMEM doorways-Mena, Iba1, endomucin) and immunofluorescence (VEGF-A (green), F4/80 (white), endomucin (red), and DAPI (blue)). TMEM doorways were identified as described in Fig. 1B. The circle in the IHC (black) and IF (white) panels show the same TMEM doorways obtained from the alignment of serial sections, and the three cells making up the TMEM doorway are indicated with the yellow triangle in each panel (TM, TEC, TTC). The TMEM doorway macrophage (TM) in the IF image, stained with F4/80 (white), is outlined in the rightmost panel. Scale bars = 20 µm. **G** The F4/80 (white) in the IF-stained slide within the TMEM doorway circle ROI was used to identify TMEM doorway macrophages and the immunofluorescence intensity of VEGF-A expression (green) within the F4/80 TMEM doorway macrophage was quantified. *n* = 5 mice per group, each dot represents the average value for a mouse. *p < 0.05, analyzed by Student’s *t*-test.

To investigate if CSF-1 signaling specifically from tumor cells drives TMEM doorway opening in vivo, we utilized a patient-derived xenograft model previously described [15, 27, 28], orthotopically transplanting human HT17 tumors into *SCID* mice. Once the tumors had developed, we injected mice *i.v*. with IgG control antibodies or a human-specific CSF-1 blocking antibody, which has less than 5% blocking activity against mouse-derived CSF-1, ensuring that only signaling from human tumor-derived CSF-1 was inhibited. Twenty-four hours following injection of the blocking antibody, we injected the mice with high molecular weight dextran (155 kDa), and 1 h later, collected CTCs and sacrificed the mice (experimental model diagramed in Supplementary Fig. 3B). Acute blockade of tumor-derived CSF-1 did not affect overall macrophage density (Supplementary Fig. 3C, D). To examine the effects of blocking tumor-derived CSF-1 on TMEM doorway activity, we stained the tumor tissue for endomucin and TMR-Dextran and measured the amounts of extravascular dextran compared to the vascular area (Fig. 5A). We found that specific inhibition of tumor-derived CSF-1 decreased TMEM doorway activity as dextran extravasation was blocked (Fig. 5B). Co-localization of vascular tight junction ZO-1 and adherens junction CD31 staining increased with tumor cell CSF-1 blockade (Fig. 5C, D), providing further evidence that endothelial junctions were less permeable upon inhibition of CSF-1 signaling. We collected the blood from the mice at the time of sacrifice and found the number of CTCs was significantly lower in the mice where tumor cell CSF-1 had been inhibited compared to control mice (Fig. 5E). To determine the impact of inhibition of tumor cell-derived CSF-1 inhibition on the levels of VEGF-A expression in TMEM doorway macrophages in vivo, we stained the tumors for VEGF-A, F4/80 to identify macrophages, and endomucin. We stained a sequential tumor tissue section for TMEM doorways, aligned the two slides, and measured VEGF-A levels within TMEM doorway macrophages (Fig. 5F). We found that tumor cell-specific CSF-1 inhibition significantly increased VEGF-A levels in the TMEM doorway macrophage compared to control treated mice (Fig. 5G), consistent with the in vitro finding where CSF-1 signaling blockade prevented VEGF-A secretion from macrophages (Fig. 3). These in vivo findings help to uncover a mechanism for TMEM doorway-associated vascular opening (TAVO) where tumor cell secreted CSF-1 induces VEGF-A secretion from macrophages, leading to increased TMEM doorway activity, decreased vascular integrity at active TMEM doorways, and increased metastatic dissemination of tumor cells through TMEM doorways as CTCs.

## Discussion

TMEM doorway-associated vascular opening (TAVO) requires vascular endothelial growth factor-A (VEGF-A) produced by the TMEM doorway macrophage [10]. In particular, a Tie2^hi^/VEGF-A^hi^/CD206^+^/CD11b^+^/F4/80^+^ TMEM doorway-associated macrophage interacts with its associated endothelial cell through secretion of VEGF-A to mediate blood vessel opening by disrupting endothelial cell adherens and tight junctions at TMEM doorways [10]. We have demonstrated, using intravital imaging, that the vascular opening in tumors is an acute, localized, and transient event, and only occurs at TMEM doorways, as opposed to VEGF-A injected into the vasculature, which caused general and continuous leakage of blood contents from all blood vessels [10]. We have further shown that TAVO events occur simultaneously with tumor cell intravasation, and are required for tumor cell intravasation. When VEGF-A is knocked out specifically in macrophages, TAVO events are blocked, as is tumor cell intravasation [10]. These observations are consistent with and provide a mechanism for the finding that the number of TMEM doorways measured by multiplex immunohistochemistry in breast cancer sections is a clinically validated prognostic indicator of distant metastasis in breast cancer patients [12–14]. However, the signal provided by tumor cell-derived CSF-1 that triggers the secretion of VEGF-A by the TMEM doorway macrophage, the most critical step for TMEM doorway opening, has remained unknown, until now. We found that CSF-1 levels within the TMEM doorways is strongly correlated (Fig. 1E, *r* = 0.9733) with extravascular dextran within TMEM doorways. This finding has important implications for measuring TMEM doorway activity in patient tissue sections where we currently are limited to measuring the number of TMEM doorways but have little ability to determine TMEM doorway activity or TAVO events with the lack of a fluorescent extravascular dextran marker in patient samples. TMEM doorways score is prognostic for distant recurrence in patients with ER^+^/HER2^−^ disease [12–14] and increases with neo-adjuvant chemotherapy treatment [15]. Now with the ability to measure TMEM doorway activity in patients, in future studies, we hope to extend this prognostic marker to better identify patients most at risk for relapse and allow for more informed treatment decisions.

CSF-1 drives the differentiation of myeloid precursors to macrophages and is essential for survival of macrophages [38–40]. CSF-1/CSF-1R signaling is important for tissue homeostasis, repair, and inflammation and acts as a chemoattractant for the recruitment, production, and survival of tumor-associated macrophages (TAMs) in the tumor microenvironment [41–43]. The Pollard group observed that silencing of CSF-1 specifically in breast tumor cells decreases lung metastases as well as macrophage recruitment to the primary tumor. Forced expression of CSF-1 in tumor cells causes increased lung metastasis and an increase in macrophage infiltration to the primary tumor [44]. TAMs promote tumor progression through stimulation of angiogenesis, secretion of IL-10, TGFβ, and VEGF-A, and driving of tumor cell migration, invasion, and metastasis [45–47]. As we show here, VEGF-A secretion from TMEM doorway macrophages can be triggered by tumor cell derived-CSF-1 binding to the CSF-1R on the macrophage (Figs. 2 and 3). CSF-1 signaling is known to drive invasiveness and increase metastasis, and increased expression of CSF-1 is correlated with poor prognosis in ovarian, breast, and prostate cancer patients.

**Fig. 6:**
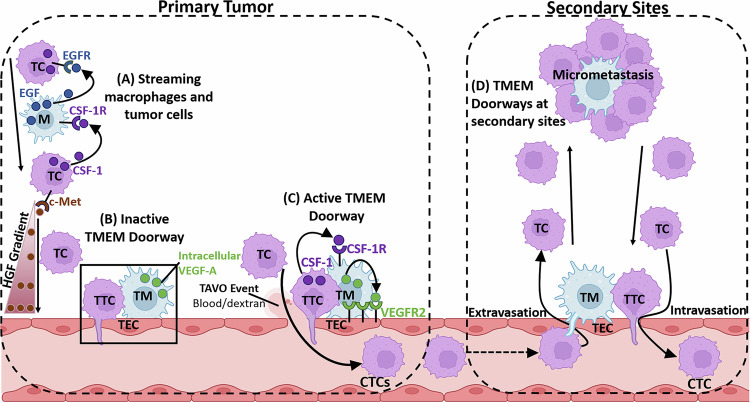
Model of CSF-1-induced TMEM doorway opening and release of CTCs into the blood stream. **A** In the primary tumor, macrophages and tumor cells exhibit a CSF-1/EGF paracrine interaction which facilitates tumor cell migration toward blood vessels in breast cancer. Tumor cells secrete CSF-1, which attracts CSF-1R-expressing macrophages, which in turn secrete EGF, increasing the migration of EGFR-expressing tumor cells. The tumor cells within the loop encounter an HGF signal found in a gradient in the tumor microenvironment with the highest concentration near blood vessels. The tumor cells migrate toward the blood vessels along the HGF gradient, adding directionality to the migration of the tumor cell-macrophage paracrine loop engaged partners. **B** In an inactive TMEM doorway (box), with the absence of any CSF-1 secreted by the TMEM doorway tumor cell (TTC), the VEGF-A remains within the TMEM doorway macrophage (TM). The blood vessels, including the TMEM doorway endothelial cell (TEC) within the tumor remain sealed and do not allow tumor cells to intravasate through TMEM doorways. **C** In contrast, in an active TMEM doorway, CSF-1 is secreted by the TMEM doorway tumor cell and binds to macrophage CSF-1R, stimulating the TMEM doorway macrophage to secrete VEGF-A. VEGF-A causes vascular opening (TAVO event) and allows tumor cells to intravasate through the TMEM doorway, creating CTCs, and dextran or blood to leak out of the vessel. **D** These circulating tumor cells (CTCs) travel through the vasculature to secondary sites where TMEM doorways are also found in metastatic foci in lymph nodes and lungs. Thin membranous connections between macrophages and tumor cells stretch from the macrophage on extravascular side of the blood vessel through the endothelial junctions and interact with CTCs, which could facilitate CTC extravasation at secondary sites [67]. TMEM doorways in metastases could also be the sites where tumor cells re-intravasate and then seed tertiary metastases. Figure created with BioRender.com.

There is evidence of a paracrine interaction between tumor cells and macrophages, which facilitates tumor cell migration toward blood vessels in breast cancer [7, 8, 48]. This interaction is driven by a CSF-1/EGF paracrine loop, wherein tumor cells secrete CSF-1 (which attracts CSF-1R-expressing macrophages), while macrophages in turn secrete EGF (which attracts EGFR-expressing tumor cells). The tumor cells respond to EGF-secreting macrophages by migrating towards them and secreting more CSF-1, therefore generating a paracrine loop [8, 26, 48, 49]. These signaling events create a chemotactic axis in which the CSF-1/EGF signals become amplified, allowing the tumor cells and macrophages to remain in close proximity with each other as they migrate together as cell pairs (a process called streaming [8, 27, 29, 36]). Once the tumor cells within the loop encounter a Hepatic Growth Factor (HGF) signal, which is found in a gradient in the tumor microenvironment with the highest concentration near blood vessels [7], the tumor cell-macrophage pairs start migrating toward the blood vessels along the HGF gradient, adding directionality to the streaming cell pairs (Fig. 6A). Once at the blood vessels, the streaming tumor cells can intravasate through active TMEM doorways, following a TAVO event [7, 10, 11]. The genetic or pharmacological inhibition of EGF, CSF-1, or the HGF receptor (c-Met) significantly impairs streaming of both tumor cells and macrophages and blocks tumor cell dissemination in in vivo breast cancer metastasis models, confirming the critical role of the paracrine CSF-1/EGF loop between tumor cells and macrophages, as well as the HGF gradient, in metastasis.

Here we demonstrate that while the TMEM doorway tumor cell and macrophage remain in direct and stable contact on the blood vessel (Fig. 6B) [10, 13], the CSF-1 signaling persists, resulting in localized macrophage secretion of VEGF-A. We found that TMEM doorway tumor cells secrete CSF-1, which binds to the CSF-1R on TMEM doorway macrophages (Fig. 6C). We further show that this event triggers localized secretion of VEGF-A by TMEM doorway macrophages and leads to downstream TAVO events, where acute vascular opening within the endothelium allows tumor cells to intravasate through the TMEM doorway. Previously, we have shown that the resulting CTCs can form distant metastases (Fig. 6C, D) [10, 15, 50]. In this study, we demonstrate that inhibition of CSF-1R signaling in vitro by either knocking out CSF-1R in macrophages, or adding a blocking antibody, decreased macrophage release of VEGF-A, as well as trans-endothelial migration of tumor cells, an in vitro measure of intravasation capability. In vivo, acute treatment of tumor-bearing mice with a CSF-1R blocking antibody significantly increased vascular tight junction stability, decreased TMEM doorway activity and TAVO events, as well as formation of CTCs. To determine whether tumor cells signal through CSF-1/CSF-1R to elicit secretion of VEGF-A from TMEM doorway macrophages and set off the cascade of events leading to TMEM doorway opening and tumor cell dissemination in vivo, we utilized a human-specific CSF-1 blocking antibody in a PDX model of breast cancer. In this model, we could isolate and block the tumor-derived CSF-1, as the tumor cells are the only human cells in the model and any CSF-1 produced by the mouse (e.g., macrophages, monocytes, endothelial cells, fibroblasts) is not inhibited. As in the CSF-1R blocking antibody experiments (Fig. 4A–D), blocking tumor-derived CSF-1 inhibited TMEM doorway activity, increased vascular integrity, and decreased dissemination of tumor cells as CTCs (Fig. 5A–E). We further found that blocking tumor-derived CSF-1 increased the levels of VEGF-A staining within TMEM doorway macrophages (Fig. 5F, G), consistent with the results from in vitro experiments demonstrating that CSF-1 signaling can induce VEGF-A secretion from macrophages and that blocking CSF-1 signaling causes macrophages to retain VEGF-A intracellularly (Fig. 3). This finding could be crucial for the ability to reduce the effects of VEGF-A signaling in the TME without relying on anti-VEGF-A therapies, discussed later.

The importance of this study beyond the primary tumor is emphasized by the finding that TMEM doorways are found not only in invasive ductal breast carcinomas, but also in metastatic foci in lymph nodes [16, 17] and lungs [6]. We and others have found that secondary metastases can seed tertiary metastases [1–4], and the presence of TMEM doorways at secondary sites may perpetuate metastatic dissemination (Fig. 6D) even after removal of the primary tumor. Therefore, blocking the function of TMEM doorways could not only prevent the dissemination of cancer cells from primary tumors, but also prevent re-dissemination of tumor cells from metastatic sites, which may occur after the resection of primary tumors [10, 11, 51, 52]. Importantly, even if the tumors are highly metastatic, we may be able to increase patient survival or quality of life, or both, by blocking TMEM doorway function systemically, which would lessen overall metastatic burden. Inhibition of both Tie2 and CSF-1 signaling to prevent TAVO events, along with standard of care therapies, might be an effective way to prevent re-dissemination from both primary and metastatic tumor sites, and allow for better patient survival.

Recent preclinical studies targeting CSF-1/CSF-1R have yielded promising results in several tumor models. A monoclonal antibody targeting CSF-1R depleted TAMs and increased the CD8^+^/CD4^+^ T cell ratio, leading to decreased primary tumor burden and decreased metastasis in mouse models of colorectal cancer and fibrosarcoma [53]. Another recent study investigating breast and colon carcinoma models found that CSF-1/CSF-1R signaling inhibition stimulated the expansion of neo-epitope-specific T cells and promoted the development of an immune-permissive TME [54]. So far, the FDA has approved two CSF-1R inhibitors for patients with tenosynovial giant cell tumors, a tumor type characterized by chromosomal translocations of the CSF-1 gene, leading to aberrant CSF-1 expression [55–57]. Despite the promising preclinical studies using CSF-1/CSF-1R inhibitors in combination with chemotherapy, immunotherapy, or radiotherapy, phase II clinical trials have not yielded the same anti-tumor efficacies [58–64]. In patients with advanced triple negative breast cancer, the combination of a CSF-1 monoclonal antibody with gemcitabine and carboplatin showed comparable anti-tumor activity and progression-free survival compared to gemcitabine and carboplatin alone [65]. In another phase Ib/II study, patients with advanced pancreatic ductal carcinoma, colorectal cancer, or non-small cell lung cancer were treated with a CSF-1R monoclonal antibody plus anti-PD-1 therapy; however, minimal anti-tumor activity was observed [62]. In these studies, investigators primarily focused on effects on primary tumor progression and were expecting to observe an enhancement in intratumoral infiltration of CD8^+^/CD4^+^ T cells and TAM depletion, given the role that CSF-1 signaling plays in creating an immune suppressive environment and promoting recruitment, survival and differentiation of macrophages. Taking into consideration our findings that CSF-1 signaling is critical for the recruitment of tumor cell-macrophage pairs to the blood vessels in the primary tumor, the transient opening of the blood vessels at TMEM doorways (Fig. 4B), and intravasation of tumor cells at TMEM doorways (Figs. 4D and 5E), we expect that treating patients with CSF-1R inhibitors would be efficacious in preventing tumor cell dissemination, metastatic disease and therefore reducing metastatic burden, though not necessarily in preventing primary tumor growth. Additionally, given our findings that TMEM doorway scores are predictive of distant metastasis in subsets of breast cancer patients [12–14], we contend that selecting the breast cancer patients meeting the criteria for predictive metastatic disease, including TMEM doorway score, for the clinical trials would show increased success in improving patient outcomes. Our study provides evidence that the mechanism through which CSF-1R inhibition can prevent metastasis is not only by macrophage depletion, but also by blocking CSF-1/CSF-1R paracrine signaling between tumor cells and macrophages leading to inhibition of tumor cell intravasation at TMEM doorways in the primary tumor. Future studies should include inhibition of tumor cell dissemination as a primary end point to evaluate the value of treatment with CSF-1R inhibitors.

We have previously shown in mice that transient, short-term blockade of VEGF-A expression in macrophages is effective in inhibiting TMEM doorway activity, TAVO events, and formation of CTCs [10]. However, if patients are treated with anti-angiogenic therapies targeting VEGF-A (e.g., bevacizumab and other VEGF-A inhibitors) they eventually develop therapeutic resistance [66]. One mechanism of tumor resistance or recurrence following anti-angiogenic therapy in patients is attributed to the increased recruitment and infiltration of Tie2-expressing macrophages into the tumor in response to apoptosis, necrosis, and hypoxia after vascular regression [66]. Additionally, the ligand for Tie2, Ang2, is upregulated in response to hypoxic conditions. Tie2-Ang2 signaling can function in a similar manner to VEGF-A signaling to promote tumor angiogenesis, bypassing VEGF-A inhibition, and even being enhanced by such inhibition. As such, Tie2 is an attractive pharmacological target for the suppression of tumor angiogenesis and tumor cell dissemination. We have found that TMEM doorway macrophages are also characterized by expression of Tie2 [10] and that a selective inhibitor of Tie2, rebastinib, inhibits TMEM doorway activity, formation of CTCs, and metastasis in several mouse models of metastasis and patient-derived xenografts [30]. The mechanism of how Tie2 contributes to signaling between the TMEM doorway cells to mediate the TAVO events is still unclear and warrants further investigation. Uncovering the complete mechanism of TMEM doorway opening in primary and secondary sites is critical. We expect that inhibiting TAVO events, potentially by targeting either or both CSF-1 and Tie2 signaling, will improve our ability to increase the survival of both patients with metastatic disease and patients who are likely to develop metastatic disease.

## Supplementary information

Supplemental Figures and Legends
