# Supplementary material for: Targeting CSF-1 signaling between tumor cells and macrophages at TMEM doorways inhibits breast cancer dissemination
Source: Oncogene. 2025 Jul 11;44(36):3297–309. doi: 10.1038/s41388-025-03485-y (PMC12399421; doi:10.1038/s41388-025-03485-y)

**Supplemental Figure Legends:**

**Supplemental Figure 1. CSF-1 is required for bone marrow derived macrophage VEGF-A secretion and tumor cell secreted CSF-1 increases macrophage VEGF-A secretion. A)** ELISA determination of VEGF-A (pg/mL) in the medium conditioned by macrophages (BMMs) co-cultured with or without tumor cells (MDA-MB-231), with medium conditioned by MDA-MB-231 tumor cells (tumor cell CM), or in presence of CSF-1R small molecule inhibitor (GW2580, CSF-1R inhib.) or vehicle (DMSO). n=3 individual experiments performed in duplicate, ****p<0.0001 by two-way ANOVA. **B)** 4T1 tumor cells were cultured for 24 hours in serum-free media and the concentration of CSF-1 (pg/mL) secreted by the 4T1 cells was measured in the tumor cell conditioned media by ELISA. Control is media not exposed to tumor cells but treated in the same way as the cells. n=3, ***p<0.001, analyzed by Student's *t*-test. **C)** ELISA determination of VEGF-A concentration (pg/mL) in medium conditioned by macrophages (BAC1.2F5) co-cultured with 4T1 tumor cells treated with control antibody (Ctrl Ab), CSF-1R blocking Ab (CSF-1R Ab), CSF-1R inhibitor (GW2580, CSF-1R inhib.) or DMSO control. n=2 individual experiments done in duplicate, *p<0.05, ***p<0.001, ****p<0.0001 analyzed by two-way ANOVA. **D)** Quantification of the immunofluorescence staining intensity of VEGF-A in macrophages (labelled with CellTracker$^{TM}$ Green) cultured with or without 4T1 tumor cells (labelled with CellTracker$^{TM}$ Red) and treated with either ctrl Ab or CSF-1R blocking Ab. The amount of VEGF-A in the macrophage was quantified using ImageJ. n=3 independent experiments, at least 57 cells analyzed per treatment group, each dot represents one cell. ****p<0.0001 analyzed by one-way ANOVA. **E)** THP-1 human-derived monocytes were treated for 48 hours with 100 ng/mL PMA to differentiate the cells into macrophages. PMA was then removed and replaced with normal media. Cells were then fixed with 2% paraformaldehyde, permeablized and stained with or without (2º only) antibodies against VEGF-A (green) and with DAPI (blue). Images show the THP-1 cells express VEGF-A. THP-1 cells were differentiated as described here for ELISA experiments in panels F-

G. **F)** ELISA determination of VEGF-A (pg/mL) in the medium conditioned by macrophages (THP-1 cells differentiated into macrophages) co-cultured with or without tumor cells (MDA-MB-231), in the presence of CSF-1R blocking antibodies (CSF-1R Ab) or isotype control (Control Ab), and in presence of CSF-1R small molecule inhibitor (GW2580, CSF-1R inhib.) or vehicle (DMSO). As both the tumor cells and macrophages are of human origin and secrete VEGF-A which is detected by the ELISA antibodies, in order to isolate the concentration of VEGF-A secreted by macrophages, we collected media from tumor cells incubated separately at each time point and subtracted this VEGF-A concentration from the total VEGF-A concentration determined by the ELISA. n=3 individual experiments performed in duplicate, ****p<0.0001, ***p<0.001 by two-way ANOVA. **G)** ELISA determination of VEGF-A (pg/mL) performed as described in (F), except using medium conditioned MDA-MB-231 tumor cells (tumor cell C.M.) instead of tumor cell co-culture. Here, to correct for tumor cell secreted VEGF-A, the concentration of VEGF-A in the MDA-MB-231 C.M., prior to adding to THP-1 cells, was determined and subtracted from all treatment groups where the tumor cell C.M. conditioned media was used. n=3 individual experiments performed in duplicate, **p<0.01 by two-way ANOVA.

**Supplemental Figure 2. CSF-1 secreted by tumor cells does not increase macrophage VEGF-A mRNA levels and schematic for CSF-1R inhibition *in vivo*. A)** Fold change VEGF-A mRNA expression, determined by qPCR, in macrophages (Mø, BAC1.2F5) co-cultured with tumor cells (TC, MDA-MB-231) compared to macrophages cultured alone, without tumor cells. Murine VEGF-A mRNA expression was normalized to murine GAPDH mRNA expression, as the endogenous control. n=3 individual experiments performed in triplicate, non-significant (ns), analyzed by Student's *t*-test. **B)** Schematic of *in vivo* experiment from Figure 4A-D where tumor pieces from *MMTV-PyMT* mice were orthotopically transplanted into syngeneic FVB mice and tumors were allowed to grow up to 1.5 $cm^3$. Four hours prior to sacrifice, mice were injected *i.v.* with 2.5 µg isotype control or blocking antibodies against CSF-1R. One hour prior to sacrifice,

mice were injected *i.v.* with 20 mg/mL 155 kDa TMR-dextran. Mice were then anesthetized, and blood was collected via cardiac puncture to collect circulating tumor cells (CTCs), and primary tumors were harvested.

**Supplemental Figure 3. CSF-1 knockdown in MDA-MB-231 cells and schematic for CSF-1 inhibition *in vivo*. A)** Fold change CSF-1 mRNA expression, determined by qPCR, in MDA-MB-231 cells transfected using Lipofectamine 2000 with ctrl siRNA or 3 different CSF-1 targeting siRNAs (#1, #2, #3) for 48hrs. Ctrl siRNA CSF-1 expression was set to one and all other treatments were set relative to this control. CSF-1 mRNA expression was normalized to GAPDH mRNA expression, as the endogenous control, in all treatment groups. n=3 individual experiments performed in triplicate, ***$p<0.001$, ****$p<0.0001$ analyzed by one-way ANOVA. **B)** Schematic of *in vivo* experiment from Figure 5 where human HT17 PDX tumor pieces were orthotopically transplanted into *SCID* mice. Tumors were allowed to grow up to 1.5 $cm^3$. Twenty-four hours prior to sacrifice, mice were injected *i.v.* with 5 µg isotype control or blocking antibodies against human CSF-1. One hour prior to sacrifice, mice were injected *i.v.* with 20 mg/mL 155 kDa TMR-dextran. Mice were then anesthetized, and blood was collected via cardiac puncture to collect circulating tumor cells (CTCs), and primary tumors were harvested. **C)** Tumor tissue from mice treated with control or CSF-1 blocking antibody, from (B) and Figure 5, were stained with F4/80 (green) and DAPI (blue). **D)** Macrophage density quantified by measuring F4/80 positive area and normalizing to DAPI positive area from staining in (C). n=5 mice per treatment group, each dot represents the average value for a mouse, ns= not significant, analyzed by Student's *t*-test.

## Supplemental Figure 1

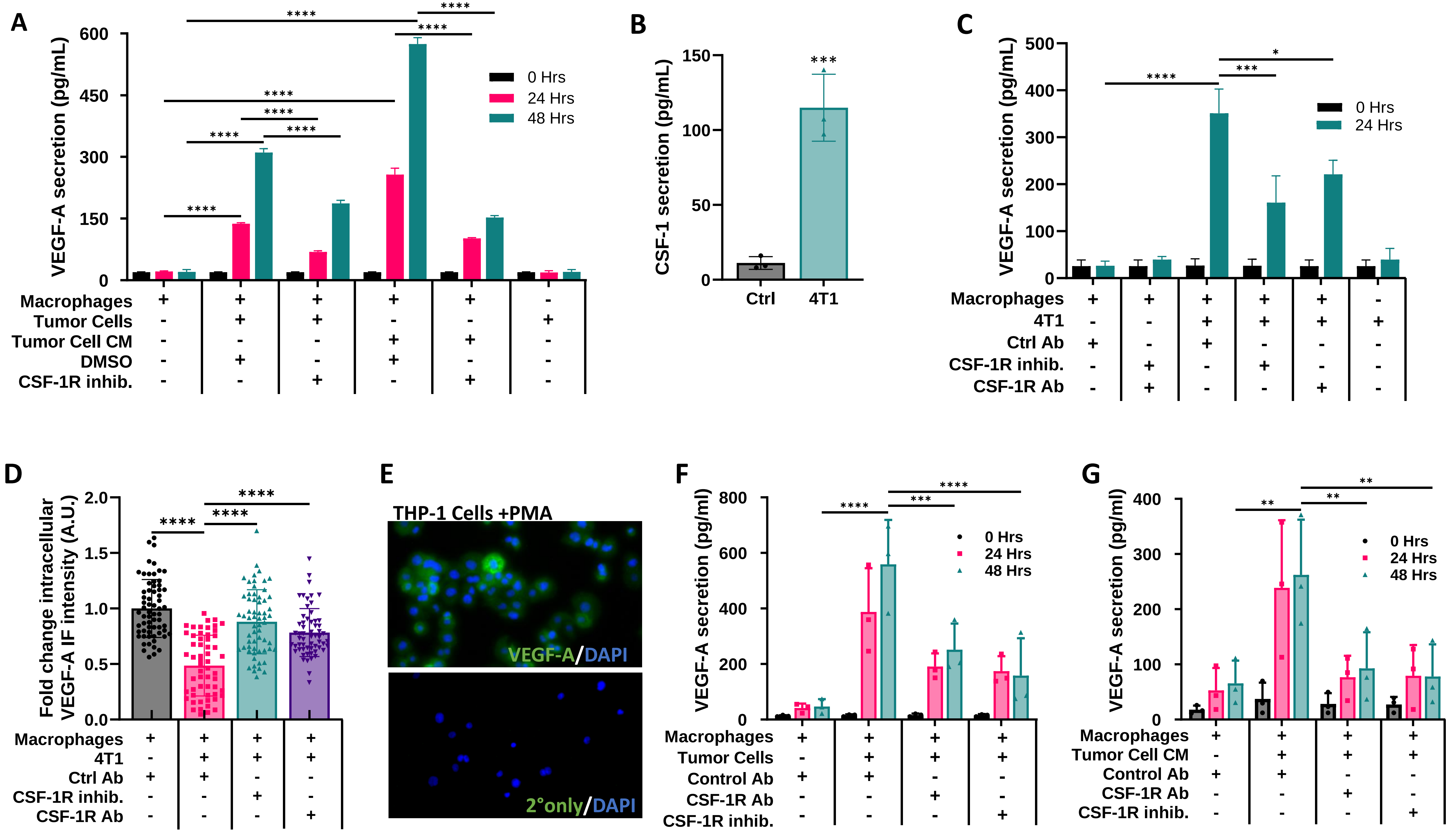

## Supplemental Figure 2

A

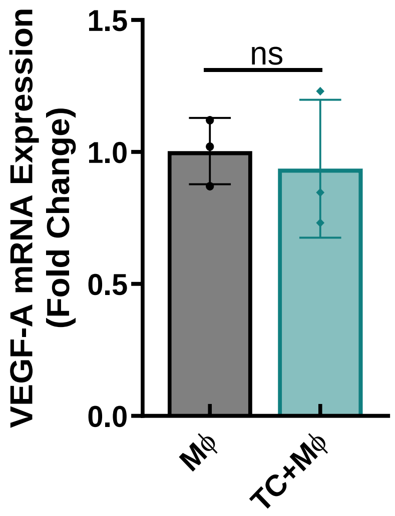


B

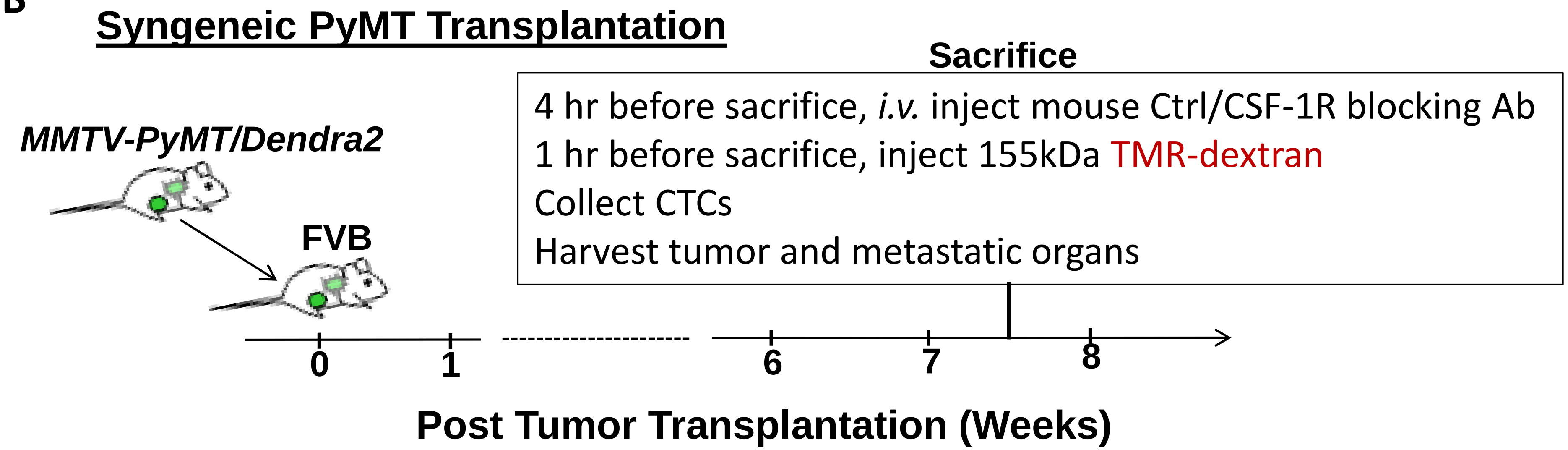

**Supplemental Figure 3**

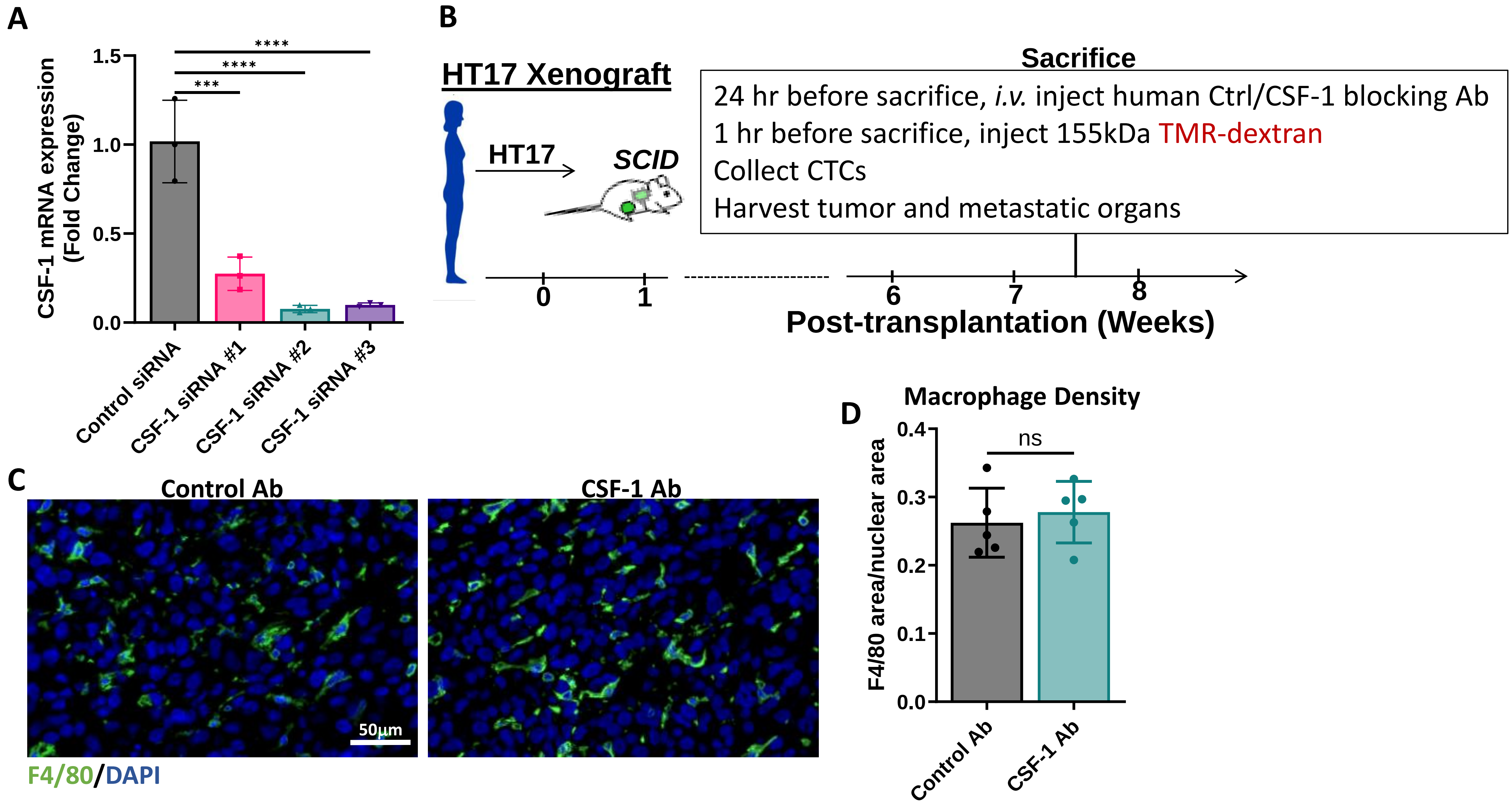

Supplement: Supplementary file 1 — Supplemental Figures and Legends [file 41388_2025_3485_MOESM1_ESM.pdf]
